# 2-Amino-3-nitro­pyridinium perrhenate

**DOI:** 10.1107/S160053681001812X

**Published:** 2010-05-22

**Authors:** Zeid Abdellah Al Othman, Samah Toumi Akriche, Mohamed Rzaigui, Refaat Mohamed Mahfouz

**Affiliations:** a Chemistry Department, Faculty of Science, King Saud University, PO Box 2455, Riyadh 11451, Saudi Arabia; b Laboratoire de Chimie des Matériaux, Faculté des Sciences de Bizerte, 7021 Zarzouna Bizerte, Tunisia

## Abstract

In the title mol­ecular salt, (C_5_H_6_N_3_O_2_)[ReO_4_], the cations and tetrahedral anions are linked by trifurcated N—H⋯(O,O,O) and bifurcated N—H⋯(O,O) hydrogen bonds, as well as weak C—H⋯O inter­actions. This results in alternating corrugated inorganic and organic layers in the crystal.

## Related literature

For hydrogen-bond inter­actions see: Katayev *et al.* (2006 ▶). For related structures containing 2-amino-3-nitro­pyridinium cations, see: Akriche & Rzaigui (2000 ▶, 2009 ▶); Toumi Akriche *et al.* (2010 ▶). For related structures containing perrhenate anions, see: Rodrigues *et al.* (2009 ▶); Ray *et al.* (2002 ▶, 2003 ▶). For distortion indices, see: Baur (1974 ▶).
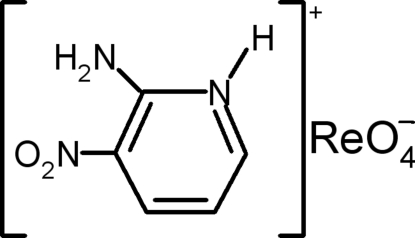


## Experimental

### 

#### Crystal data

(C_5_H_6_N_3_O_2_)[ReO_4_]
*M* *_r_* = 390.33
Monoclinic, 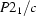

*a* = 6.235 (3) Å
*b* = 22.030 (2) Å
*c* = 7.840 (6) Å
β = 117.52 (5)°
*V* = 955.0 (9) Å^3^
*Z* = 4
Ag *K*α radiation
λ = 0.56087 Å
μ = 6.86 mm^−1^
*T* = 293 K
0.50 × 0.40 × 0.30 mm

#### Data collection

Enraf–Nonius CAD-4 diffractometer
Absorption correction: multi-scan (Blessing, 1995 ▶) *T*_min_ = 0.054, *T*_max_ = 0.134
7565 measured reflections
4682 independent reflections
3532 reflections with *I* > 2σ(*I*)
*R*_int_ = 0.027
2 standard reflections every 120 min  intensity decay: 4%

#### Refinement

*R*[*F* ^2^ > 2σ(*F* ^2^)] = 0.040
*wR*(*F* ^2^) = 0.111
*S* = 1.07
4682 reflections
137 parameters
24 restraints
H-atom parameters constrained
Δρ_max_ = 2.71 e Å^−3^
Δρ_min_ = −2.35 e Å^−3^

### 

Data collection: *CAD-4 EXPRESS* (Enraf–Nonius, 1994 ▶); cell refinement: *CAD-4 EXPRESS*; data reduction: *XCAD4* (Harms & Wocadlo, 1995 ▶); program(s) used to solve structure: *SHELXS97* (Sheldrick, 2008 ▶); program(s) used to refine structure: *SHELXL97* (Sheldrick, 2008 ▶); molecular graphics: *ORTEP-3* (Farrugia, 1997 ▶) and *DIAMOND* (Brandenburg & Putz, 2005 ▶); software used to prepare material for publication: *WinGX* (Farrugia, 1999 ▶).

## Supplementary Material

Crystal structure: contains datablocks I, global. DOI: 10.1107/S160053681001812X/hb5451sup1.cif

Structure factors: contains datablocks I. DOI: 10.1107/S160053681001812X/hb5451Isup2.hkl

Additional supplementary materials:  crystallographic information; 3D view; checkCIF report

## Figures and Tables

**Table 1:** Selected bond lengths (Å)

Re1—O1	1.726 (6)
Re1—O2	1.706 (7)
Re1—O3	1.708 (8)
Re1—O4	1.665 (7)

**Table 2:** Hydrogen-bond geometry (Å, °)

*D*—H⋯*A*	*D*—H	H⋯*A*	*D*⋯*A*	*D*—H⋯*A*
N1—H1⋯O1	0.86	2.37	3.041 (10)	135
N1—H1⋯O2^i^	0.86	2.42	3.018 (10)	128
N1—H1⋯O1^ii^	0.86	2.48	3.077 (11)	128
N2—H2*A*⋯O1	0.86	2.38	3.036 (10)	133
N2—H2*A*⋯O1^ii^	0.86	2.40	3.010 (10)	129
N2—H2*A*⋯O2^iii^	0.86	2.55	3.168 (11)	129
N2—H2*B*⋯O5	0.86	2.02	2.624 (11)	127
N2—H2*B*⋯O3^iii^	0.86	2.26	2.976 (12)	141
C3—H3⋯O5^iv^	0.93	2.44	3.138 (12)	132
C5—H5⋯O4^v^	0.93	2.32	3.094 (13)	141
C5—H5⋯O2^i^	0.93	2.41	3.020 (13)	123

Symmetry codes: (i) 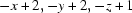; (ii) 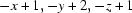; (iii) 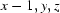; (iv) 
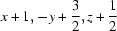
; (v) 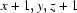.

**Fig. 1.**
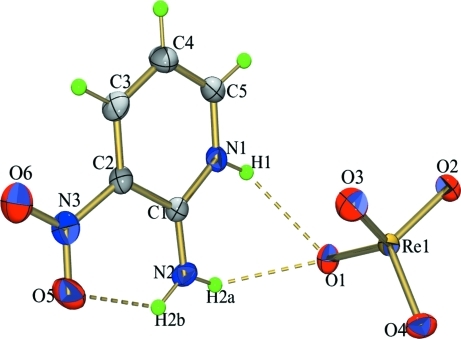
A view of (I) with displacement ellipsoids drawn at the 30% probability level. Hydrogen bonds are represented as dashed lines.

**Fig. 2.**
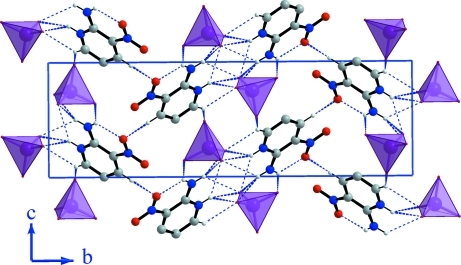
Projection of (I) along the a axis. The H-atoms not involved in H-bonding are omitted.

## supplementary crystallographic information

### Comment

A new engineering strategy aimed at building very cohesive frameworks based on oxyanion subnetworks in which the organic molecules are strongly anchored thanks to different interactions (electrostatic, H-bonds, Van der Waals). These interactions are all important in the construction of atomic arrangement but short and multiple hydrogen-bonds observed in these frameworks appear to be the most exciting since they have been recognized as the steering force responsible for the formation of special networks (Katayev *et al.*, 2006). The oxoanions are good hydrogen bond acceptors, that's why they have been employed in the purification, extraction and detection techniques of dangerous pollutants (Ray *et al.*, 2003; Ray *et al.*, 2002; Rodrigues *et al.*, 2009). The 2-amino-3-nitropyridine molecule has a dual nature because of its donor and acceptor groups. It can be protonated and thus serve as a hydrogen bond donor and electrostatic attractive element and also serve as a hydrogen bond acceptor which is especially useful for the binding of oxoanions. In this paper, we will account on the crystal engineering of 2-amino-3-nitropyridininium perrhenate, (C_5_H_6_N_3_O_2_)^+^, ReO_4_^-^ (I).

The asymmetric unit of (I) consists of one perrhenate anion (ReO_4_^-^ ) and one organic cation (C_5_H_6_N_3_O_2_)^+^ (Fig. 1).

The atomic arrangement of this salt is an organized dispersion of oganic cations and inorganic anions which form alternate corrugated layers (Fig. 2). This projection shows too the extensive network of H-bonds, N—H···O and C—H···O between cation and anion and C—H···O between cations (Table 1). These interactions constitute a key factor as well as electrostatic interaction in the stabilization of this structure. The nitropyridinium cations form chains running parallel to the [101/2] direction. The C—H···O interactions have the same H-bonds geometric parameters as the N—H···O ones which may also consolidate the cohesion of this structure. The neighbour nitropyridinium cations, are linked to form one-dimensional chains via C3—H3···O5 H-bonds (see Table 1 for symmetry codes) with C···O distance of 2.44 Å. Such chains of 2-amino-3-nitropyridinium are also observed in the related structure of 2 A3NPClO4 (Toumi Akriche *et al.*, 2010). In this structure, the (C_5_H_6_N_3_O_2_)^+n^ chains connect the discrete ReO_4_^-^ anions through N—H···O and C—H···O hydrogen bonds in all directions to develop a three-dimensional network. It's worth noticing that the particular behaviour of hydrogen of nitrogen atoms which establish bi- and trifurcated H-bonds, that well explains the weak values of the corresponding angles spreading between 123 and 141°.

The ReO_4_^- ^anion have an expected but slightly distorted tetrahedral geometry around Re atom with the Re—O bond lengths ranging from 1.665 (7) to 1.726 (6) Å and the O—Re—O bond angles ranging from 107.4 (4) to 113.4 (4)°. The average Re—O bond distances and O—Re—O bond angles are 1.701 Å and 109.45°, respectively, confirming a tetrahedral configuration, similar to other studied perrhenates (Ray *et al.*, 2003; Ray *et al.*, 2002). Nevertheless, the calculated average values of the distortion indices (Baur *et al.*, 1974) corresponding to the different angles and distances in the independent ReO_4_ tetrahedron (DI(Re—O) = 0.011, DI(O—Re—O) = 0.017, and DI( O—O ) = 0.013) show an above distortion of the O—O distances compared to Re—O distances. The same feature is observed in ClO_4_ tetrahedron of the related structure of 2-amino-3-nitropyridinium perchlorate (Toumi Akriche *et al.*, 2010). However, the distortion indices observed in 2-amino-3-nitropyridinium phosphate and selenate structures show an above distortion of the X—O (X = P and Se) distances compared to O—O distances (Akriche *et al.*, 2000; Akriche *et al.*, 2009), that well explain the distortion of XO_4_ tetrahedra (X = P and Se) in which the P and Se atoms are displaced of 0.114 to 0.065 Å from gravity center of XO_4_. The ReO_4_ tetrahedron is thus described by a regular oxygen atoms arrangement with the rhenium atom slightly shifted from gravity center of ReO_4_ (0.042 Å).

As expected, the pyridinium ring of 2-amino-3-nitropyridinium cation is nearly planar, with maximum deviation from planarity equal to 0.021 (5) Å. The diedral angle between the planes of the NO_2 _group and the pyridinium ring is close to 4.25 (9)° indicating a deformation of the NO_2_ group since the oxygen atoms of this later are the seat of various types of inter-and intramolecular hydrogen bonds. The geometrical charcteristics of the (I) organic cation are normal and comparable to that observed for the same species in other structure (Akriche*et al.*, 2000; Toumi Akriche *et al.*, 2010; Akriche *et al.*, 2009 ).

### Experimental

An aqueous solution (10 ml) of NH_4_ReO_4_ (0.27 g; 1 mmol) is added drop by drop under stirring to an ethanolic solution (5 ml) of 2-amino-3-nitropyridine (0,139 g; 1 mmol) in the presence of HCL (1 M, 1 ml). Yellow solution was left in air for a week until yellow prisms of (I) were deposited on the wall of the beaker.

### Refinement

All H atoms attached to C and N atoms were fixed geometrically and treated as riding, with C—H = 0.93 Å and N—H = 0.86 Å and with *U*_iso_(H) = 1.2Ueq(C or N).

### Crystal data

**Table:**

(C_5_H_6_N_3_O_2_)[ReO_4_]	*F*(000) = 720
*M**_r_* = 390.33	*D*_x_ = 2.715 Mg m^−^^3^
Monoclinic, *P*2_1_/*c*	Ag *K*α radiation, λ = 0.56087 Å
Hall symbol: -P 2ybc	Cell parameters from 25 reflections
*a* = 6.235 (3) Å	θ = 9–11°
*b* = 22.030 (2) Å	µ = 6.86 mm^−^^1^
*c* = 7.840 (6) Å	*T* = 293 K
β = 117.52 (5)°	Prism, yellow
*V* = 955.0 (9) Å^3^	0.50 × 0.40 × 0.30 mm
*Z* = 4	

### Data collection

**Table:**

Enraf–Nonius CAD-4 diffractometer	3532 reflections with *I* > 2σ(*I*)
Radiation source: Enraf–Nonius FR590	*R*_int_ = 0.027
graphite	θ_max_ = 28.0°, θ_min_ = 2.4°
non–profiled ω scans	*h* = −10→10
Absorption correction: multi-scan (Blessing, 1995)	*k* = −36→0
*T*_min_ = 0.054, *T*_max_ = 0.134	*l* = −12→13
7565 measured reflections	2 standard reflections every 120 min
4682 independent reflections	intensity decay: 4%

### Refinement

**Table:**

Refinement on *F*^2^	Secondary atom site location: difference Fourier map
Least-squares matrix: full	Hydrogen site location: inferred from neighbouring sites
*R*[*F*^2^ > 2σ(*F*^2^)] = 0.040	H-atom parameters constrained
*wR*(*F*^2^) = 0.111	*w* = 1/[σ^2^(*F*_o_^2^) + (0.052*P*)^2^ + 2.5911*P*] where *P* = (*F*_o_^2^ + 2*F*_c_^2^)/3
*S* = 1.07	(Δ/σ)_max_ = 0.037
4682 reflections	Δρ_max_ = 2.71 e Å^−^^3^
137 parameters	Δρ_min_ = −2.35 e Å^−^^3^
24 restraints	Extinction correction: *SHELXL97* (Sheldrick, 2008), Fc^*^=kFc[1+0.001xFc^2^λ^3^/sin(2θ)]^-1/4^
Primary atom site location: structure-invariant direct methods	Extinction coefficient: 0.033 (2)

### Special details

**Table:**

Geometry. All esds (except the esd in the dihedral angle between two l.s. planes) are estimated using the full covariance matrix. The cell esds are taken into account individually in the estimation of esds in distances, angles and torsion angles; correlations between esds in cell parameters are only used when they are defined by crystal symmetry. An approximate (isotropic) treatment of cell esds is used for estimating esds involving l.s. planes.
Refinement. Refinement of *F*^2^ against ALL reflections. The weighted *R*-factor wR and goodness of fit *S* are based on *F*^2^, conventional *R*-factors *R* are based on *F*, with *F* set to zero for negative *F*^2^. The threshold expression of *F*^2^ > σ(*F*^2^) is used only for calculating *R*-factors(gt) etc. and is not relevant to the choice of reflections for refinement. *R*-factors based on *F*^2^ are statistically about twice as large as those based on *F*, and *R*- factors based on ALL data will be even larger.

### Fractional atomic coordinates and isotropic or equivalent isotropic displacement parameters (Å^2^)

**Table:**

	*x*	*y*	*z*	*U*_iso_*/*U*_eq_	
Re1	0.71309 (5)	0.940210 (14)	0.25037 (4)	0.03320 (12)	
O1	0.5590 (13)	0.9742 (3)	0.3597 (10)	0.0450 (14)	
O2	0.9536 (13)	0.9832 (4)	0.2759 (11)	0.0544 (17)	
O3	0.8207 (16)	0.8717 (4)	0.3586 (14)	0.062 (2)	
O4	0.5164 (14)	0.9265 (4)	0.0220 (10)	0.0568 (19)	
O5	0.2048 (17)	0.7952 (4)	0.6686 (16)	0.073 (2)	
O6	0.453 (2)	0.7369 (5)	0.8873 (17)	0.088 (3)	
N1	0.7550 (13)	0.9105 (4)	0.7469 (10)	0.0400 (14)	
H1	0.7366	0.9443	0.6867	0.048*	
N2	0.3526 (15)	0.8922 (3)	0.5588 (11)	0.0427 (15)	
H2A	0.3378	0.9264	0.5019	0.051*	
H2B	0.2288	0.8690	0.5260	0.051*	
N3	0.4105 (19)	0.7821 (4)	0.7905 (14)	0.053 (2)	
C1	0.5584 (15)	0.8757 (3)	0.6920 (11)	0.0338 (13)	
C2	0.6059 (16)	0.8218 (4)	0.8141 (13)	0.0386 (14)	
C3	0.8316 (19)	0.8088 (4)	0.9565 (15)	0.050 (2)	
H3	0.8578	0.7733	1.0280	0.060*	
C4	1.0226 (19)	0.8481 (6)	0.9954 (18)	0.058 (3)	
H4	1.1769	0.8402	1.0937	0.070*	
C5	0.9753 (18)	0.8982 (5)	0.8848 (15)	0.050 (2)	
H5	1.1009	0.9248	0.9059	0.060*	

### Atomic displacement parameters (Å^2^)

**Table:**

	*U*^11^	*U*^22^	*U*^33^	*U*^12^	*U*^13^	*U*^23^
Re1	0.03010 (15)	0.03753 (17)	0.03126 (16)	−0.00110 (11)	0.01359 (11)	−0.00198 (11)
O1	0.057 (4)	0.041 (3)	0.045 (3)	−0.001 (3)	0.030 (3)	−0.002 (3)
O2	0.040 (3)	0.067 (5)	0.055 (4)	−0.007 (3)	0.022 (3)	0.008 (3)
O3	0.062 (5)	0.046 (4)	0.076 (6)	0.010 (3)	0.030 (4)	0.017 (4)
O4	0.044 (4)	0.078 (5)	0.039 (3)	0.001 (3)	0.012 (3)	−0.019 (3)
O5	0.057 (5)	0.057 (5)	0.090 (7)	−0.021 (4)	0.021 (4)	0.005 (5)
O6	0.090 (7)	0.059 (6)	0.103 (8)	−0.012 (5)	0.034 (6)	0.036 (5)
N1	0.041 (3)	0.037 (3)	0.046 (4)	−0.001 (2)	0.023 (3)	0.006 (3)
N2	0.046 (4)	0.035 (3)	0.037 (3)	−0.001 (3)	0.010 (3)	0.006 (3)
N3	0.064 (5)	0.031 (3)	0.064 (5)	−0.004 (3)	0.029 (4)	0.003 (3)
C1	0.039 (3)	0.028 (3)	0.034 (3)	−0.001 (2)	0.017 (3)	−0.003 (2)
C2	0.044 (4)	0.028 (3)	0.046 (4)	0.003 (3)	0.023 (3)	0.002 (3)
C3	0.054 (5)	0.038 (4)	0.057 (5)	0.014 (3)	0.024 (4)	0.017 (4)
C4	0.041 (4)	0.062 (6)	0.066 (6)	0.009 (4)	0.021 (4)	0.016 (5)
C5	0.040 (4)	0.057 (5)	0.052 (5)	−0.001 (4)	0.020 (4)	0.008 (4)

### Geometric parameters (Å, °)

**Table:**

Re1—O1	1.726 (6)	C2—C3	1.363 (13)
Re1—O2	1.706 (7)	C2—N3	1.441 (12)
Re1—O3	1.708 (8)	C2—C1	1.466 (11)
Re1—O4	1.665 (7)	O5—N3	1.228 (14)
N1—C5	1.325 (12)	N3—O6	1.206 (12)
N1—C1	1.338 (11)	C3—C4	1.387 (16)
N1—H1	0.8600	C3—H3	0.9300
N2—C1	1.277 (11)	C5—C4	1.350 (15)
N2—H2A	0.8600	C5—H5	0.9300
N2—H2B	0.8600	C4—H4	0.9300
			
O4—Re1—O2	113.4 (4)	N2—C1—N1	121.7 (8)
O4—Re1—O3	107.4 (4)	N2—C1—C2	126.0 (8)
O2—Re1—O3	108.1 (4)	N1—C1—C2	112.1 (7)
O4—Re1—O1	108.1 (4)	C3—C2—N3	118.0 (8)
O2—Re1—O1	111.2 (4)	C3—C2—C1	121.8 (8)
O3—Re1—O1	108.5 (4)	N3—C2—C1	120.3 (8)
C5—N1—C1	126.5 (8)	C2—C3—C4	120.3 (9)
C5—N1—H1	116.8	C2—C3—H3	119.9
C1—N1—H1	116.8	C4—C3—H3	119.9
C1—N2—H2A	120.0	C5—C4—C3	117.3 (10)
C1—N2—H2B	120.0	C5—C4—H4	121.3
H2A—N2—H2B	120.0	C3—C4—H4	121.3
O6—N3—O5	122.0 (10)	N1—C5—C4	121.9 (10)
O6—N3—C2	119.7 (10)	N1—C5—H5	119.1
O5—N3—C2	118.3 (8)	C4—C5—H5	119.1
			
C3—C2—N3—O6	−4.7 (15)	C2—C3—C4—C5	−1.3 (17)
C1—C2—N3—O6	177.5 (10)	C5—N1—C1—N2	179.0 (9)
C3—C2—N3—O5	176.6 (10)	C5—N1—C1—C2	4.7 (12)
C1—C2—N3—O5	−1.2 (14)	C3—C2—C1—N2	−178.3 (9)
N3—C2—C3—C4	−174.9 (10)	N3—C2—C1—N2	−0.6 (13)
C1—C2—C3—C4	2.9 (15)	C3—C2—C1—N1	−4.3 (12)
C1—N1—C5—C4	−3.5 (17)	N3—C2—C1—N1	173.5 (8)
N1—C5—C4—C3	1.4 (18)		

### Hydrogen-bond geometry (Å, °)

**Table:**

*D*—H···*A*	*D*—H	H···*A*	*D*···*A*	*D*—H···*A*
N1—H1···O1	0.86	2.37	3.041 (10)	135
N1—H1···O2^i^	0.86	2.42	3.018 (10)	128
N1—H1···O1^ii^	0.86	2.48	3.077 (11)	128
N2—H2A···O1	0.86	2.38	3.036 (10)	133
N2—H2A···O1^ii^	0.86	2.40	3.010 (10)	129
N2—H2A···O2^iii^	0.86	2.55	3.168 (11)	129
N2—H2B···O5	0.86	2.02	2.624 (11)	127
N2—H2B···O3^iii^	0.86	2.26	2.976 (12)	141
C3—H3···O5^iv^	0.93	2.44	3.138 (12)	132
C5—H5···O4^v^	0.93	2.32	3.094 (13)	141
C5—H5···O2^i^	0.93	2.41	3.020 (13)	123

Symmetry codes: (i) −*x*+2, −*y*+2, −*z*+1; (ii) −*x*+1, −*y*+2, −*z*+1; (iii) *x*−1, *y*, *z*; (iv) *x*+1, −*y*+3/2, *z*+1/2; (v) *x*+1, *y*, *z*+1.
